# Cancer in southeast Asia: can we do better?

**DOI:** 10.1016/j.lansea.2023.100216

**Published:** 2023-05-09

**Authors:** The Lancet Regional Health – Southeast Asia

In 2020, 2,252,981 new cases and 1,444,528 deaths due to cancer were reported in the WHO South-East Asia region. Among new cases, breast, cervix, and lung cancer were the most frequently reported. Economic transitions, lifestyle changes (such as physical inactivity), and rising obesity prevalence are increasing cancer risk in this region, with existing risk factors such as tobacco use and indoor and outdoor air pollution.

Although cancer is a life-threatening disease, it can be prevented and treated if detected early. Screening programmes such as breast self-examination and mammography substantially increase breast cancer survival. However, women with breast cancer are less likely to consider a mastectomy because it can be seen as a threat to femininity and sexual health, leading to body image issues, stress, and anxiety. Therefore, advanced options, such as breast-conserving surgery (lumpectomy) and breast reconstruction surgery, should be made more available in the South-East Asia region. Cervical cancer is the second most frequent cancer in the South-East Asia region caused by high-risk human papillomavirus (HPV) and can be prevented by vaccination. Bhutan introduced the HPV vaccine as part of the national immunisation programme in 2010, whereas India only rolled out the vaccination programme for girls aged 9–14 years from January, 2023. Increasing HPV vaccination awareness is important, and boys also need to be included in the national HPV immunisation programmes because this virus can also cause cancer of the mouth and throat, penis, or anus.

Tobacco is another major cause of cancer and preventable deaths worldwide. More than 22% of adult smokers are from the South-East Asia region, in addition to being the majority (81%) of smokeless tobacco users. Strengthening tax administration and increasing the prices are effective strategies to curb illicit tobacco trade and tobacco use. India has shown that political will can lead to transformative impact in implementing policy. However, more needs to be done as tobacco and smoking are still the cause of many deaths in the region.

Equal attention should be paid to less prevalent types of cancer. Mailankody and colleagues studied 30 population-based cancer registries in India and national registries in Sri Lanka, Bhutan, and Nepal. Most incident cancers (>60%) were rare, except those in Sri Lanka. With such high burden of disease in the region there is a huge need for not only availability of affordable and accessible treatment but also preventive services and screening programmes for early detection and timely treatment of cancer. The field would also benefit from research on defined treatment protocols for local populations. Most patients spend out-of-pocket for treatment in private facilities because public facilities are overloaded and rural health systems are fragmented to proper referrals. This issue often leads to financial toxicity and catastrophic expenditures. Governments need to include cancer treatment in national insurance—not restricted to surgeries but allowing lifelong treatment. In Indonesia, coverage for cancer treatment has been included in national insurance since 2014.

There is a paucity of research on the effects of treatments in individuals with cancer in the South-East Asia region, particularly well designed, multicentre, and statistically powered randomised controlled trials. In addition, there are no clear guideline updates that would be in line with international standards. This limitation creates a wide gap in providing effective treatment and services to patients in resource-limited settings, such as most countries in the South-East Asia region. To tackle such differences, the Pan-Asian Guidelines Adaptation (PAGA) project of the European Society for Medical Oncology has collaborated with oncology societies in some Asian countries. Currently, only three of ten countries in the South-East Asia region are involved in the PAGA project. There is a need for continued effort from clinicians, researchers, and policy makers to collaborate and find the most effective strategies to serve patients in the best way possible.

